# Plasticity in the prefrontal cortex of adult rats

**DOI:** 10.3389/fncel.2015.00015

**Published:** 2015-02-03

**Authors:** Bryan Kolb, Robbin Gibb

**Affiliations:** Canadian Centre for Behavioural Neuroscience, University of LethbridgeLethbridge, AB, Canada

**Keywords:** prefrontal cortex, experience-dependent plasticity, psychoactive drugs, stress, metaplasticity

## Abstract

We review the plastic changes of the prefrontal cortex of the rat in response to a wide range of experiences including sensory and motor experience, gonadal hormones, psychoactive drugs, learning tasks, stress, social experience, metaplastic experiences, and brain injury. Our focus is on synaptic changes (dendritic morphology and spine density) in pyramidal neurons and the relationship to behavioral changes. The most general conclusion we can reach is that the prefrontal cortex is extremely plastic and that the medial and orbital prefrontal regions frequently respond very differently to the same experience in the same brain and the rules that govern prefrontal plasticity appear to differ for those of other cortical regions.

Most knowledge about plasticity in the prefrontal cortex comes from studies of rodents, although there is obviously an extensive literature on other brain-behavior relationships in many other species, especially monkeys and humans. The rodent studies can inform studies of primates although direct homologies are controversial and likely impossible. In the 1960s the prefrontal cortex was defined both by the frontal granular cell layer and by the connections with the dorsomedial nucleus of the thalamus (MD) (see volume by Rose and Woolsey, 1948; Warren and Akert, 1964). One problem is that although the MD-projection cortex and granular cortex overlap, the MD-projection cortex extends well beyond the granular regions in primates (see Wise, 2008). Although rodents do not have frontal granular cortex, Leonard (1969, 1972) described frontal regions of the rat receiving projections from MD and she called this tissue “prefrontal cortex.” Contrary to popular belief at the time, this region did not include the frontal pole but rather included regions along the anterior medial wall of the cerebral hemispheres as well as the ventral and lateral regions bordering the rhinal fissure (see Figure 1). Later rodent prefrontal definitions expanded to include the connections with amygdala and ventral tegmentum (Reep, 1984; Schoenbaum and Setlow, 2002) as well as basal ganglia (Uylings et al., 2003).

**Figure 1:**
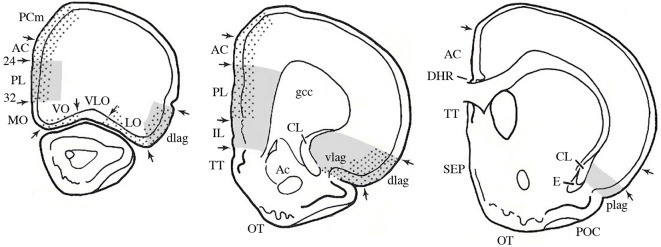
**The prefrontal cortex**. A. Serial sections through a rat brain showing different cytoarchitectonic regions. Dotted areas receive projections from MD; gray areas receive projections from the amygdala. Abbreviations: AC, anterior cingulate; CL, claustrum; gcc, genu of the corpus callousm; IL, infralimbic; lag, lateral agranular; MO, medial orbital; PL, prelimbic; plag, posterior lateral agranular; SEP, septum; TT, tania tecta; vlag, ventral lateral agranular; VLO, ventral lateral orbital; VO, ventral orbital. (Modified and adapted from Reep, 1984).

The earliest behavioral studies attempted to doubly dissociate the medial and more inferior and lateral regions by comparing the effects of lesions of the complete medial region (medial prefrontal cortex) to the orbital and insular regions (orbital frontal cortex) receiving projections from MD (e.g., Kolb et al., 1974; Kolb, 1984). In the past 40 years the lesions have become more specific to subregions (e.g., Euston et al., 2012) but the general point is the same: the medial and orbital regions have distinctly different, and complementary, functions.

But how do these regions relate to the granular cortex of primates? There is little doubt that primates have evolved frontal regions that are likely linked to the massive expansion of the cortical sensory maps (e.g., Pandya and Yeterian, 1990) and it has been argued strongly that many of these regions, and especially those forming the dorsolateral prefrontal cortex, are unique in primates (e.g., Preuss, 1995; Wise, 2008; Wallis, 2011). This is likely but it is equally likely that the primate frontal granular regions as well as other frontal regions evolved from a common ancestor that also gave rise to the rodent “prefrontal” cortex (e.g., Kolb, 2007). For the current purpose we will assume that the rodent MD-projection cortex has many similarities to the prefrontal regions of primates and has some general shared functions. Thus, studies of prefrontal plasticity in rodents should give important clues to how prefrontal regions in primates are also changed by experiences.

## Measuring experience-dependent changes in the cortex

The idea that experience modifies brain structure can be traced back at least a century although it was only in the 1960s that investigators began to study experience-dependent changes in the cortex. One fundamental assumption of these studies follows from Hebb’s (1949) idea that experience modifies the reorganization or strengthening of synaptic connections in specific neural circuits, a property usually referred to as neural plasticity. Although neural plasticity can be inferred from behavioral, electrophysiological, and molecular measures, much of the research on cortical plasticity has been on the morphology of dendrites and dendritic spines. Because our focus here is on prefrontal cortex, we will focus on changes in behavior and in synaptic organization inferred from Golgi-type analyses of changes in dendritic organization and spine density. There have been very few electrophysiological studies of rodent prefrontal cortex and most such studies are confined to the medial region. Where possible we supplement these studies with other types of information, including epigenetic changes.

It is important to note that experiences do not simply increase synapse numbers but can also reduce synapse numbers by selective pruning. Although historically there was a tacit assumption that more synapses was better, the pruning of synapses, a key element in brain development, is also related to experience-dependent behavioral changes. At our current level of understanding we really cannot do much more than demonstrate that synaptic changes have occurred and try to correlate them with behavioral changes. These changes are not always straight forward, however, as singular experiences can produce opposite changes in the synaptic density of prefrontal neurons in different prefrontal regions. Thus, synaptic change is not simply the addition of synapses but also the deletion of synapses and both types of change reflect changes in neural networks.

The neurons in the prefrontal cortex are largely excitatory pyramidal (80–90%) with the remaining neurons being inhibitory GABAergic interneurons. Both of these cell types can be further subdivided into several cell types (Ascoli et al., 2008; DeFelipe et al., 2013) but little is known about how subpopulations respond to varied experiences (but see recent review of optogenetic dissection of prefrontal cortex by Riga et al., 2014). One expanding field is related to the mechanisms maintaining an excitatory/inhibitory balance (E/I), which is likely central to how experience changes the prefrontal cortex (e.g., Yizhar et al., 2011; Kvitsiani et al., 2013).

Although the functions and anatomical organization of the rodent prefrontal cortex has been intensely studied for over 40 years, it has only been in the past decade or so that interest has shifted to the plasticity of the prefrontal regions, both in development (see review by Kolb et al., 2012) and adulthood (e.g., Robinson and Kolb, 2004; Kolb and Gibb, 2014). Because few studies have attempted to measure plasticity in subregions, we will stick with the more general terms of medial frontal (including infralimbic, prelimbic, and anterior cingulate regions) (mPFC) and orbitofrontal cortex (including orbital and insular regions) (OFC), all of which represent MD-projection cortex. We have combined the medial and orbital regions as there are few direct comparisons within subregions and we are unaware of any data showing plasticity differences within these general fields. As we consider how experiences alter synaptic organization of the prefrontal cortex, we consider eight general categories including: (1) sensory and motor experience; (2) gonadal hormones; (3) psychoactive drugs; (4) learning tasks; (5) stress; (6) social experience; (7) metaplastic experiences; and (8) brain injury.

## Sensory and motor experience

The first studies showing experience-dependent neocortical change were done by a group of researchers at Berkeley who placed rats in complex environments (sometimes called “enriched environments”) (e.g., Rosenzweig et al., 1962a). Although treated with skepticism at the time, these studies consistently showed that placing rats in such environments for a few weeks had profound, and seemingly permanent effects across the cortex including increased cortical thickness, increased dendritic arbor and complexity, and increased levels of acetylcholinesterase (e.g., Rosenzweig et al., 1962a,b; Diamond et al., 1975). Later, Greenough et al. expanded such studies to include a variety of other measures including brain size, cortical thickness, neuron size, dendritic branching, spine density, synapses per neuron, glial numbers and complexity, expression of neurotransmitters and growth factors, and vascular arborization (e.g., Sirevaag and Greenough, 1988; Greenough and Chang, 1989). But in spite of all these studies there were no studies on prefrontal areas until recently (Kolb et al., 2003b; Comeau et al., 2010), although Greenough et al. (1973) did study more lateral frontal cortex. In our initial study we were guided by the general consensus that enriched housing, which we shall call complex housing, had similar effects across the cortex including increased dendritic length and complexity, and increased spine density. We therefore compared the effects of complex housing for 3.5 months on neurons in mPFC, parietal cortex, and nucleus accumbens (NAcc) of *female* rats (Kolb et al., 2003b). The results showed that housing female rats in a complex environment increased dendritic arborization on medium spiny neurons in NAcc and on pyramidal neurons in parietal cortex but *not* in mPFC. Environmental complexity increased spine density in all regions, however. The mPFC result was surprising and led us to examine the effects of complex housing in *male* rats and in both layer III and V in mPFC, layer III in OFC, and layer III parietal cortex (Comeau et al., 2010). Layer V neurons in mPFC again showed little change in dendritic length or spine density but Layer III neurons in both mPFC and OFC showed a *decrease* in dendritic complexity and spine density, which once again was unexpected. Parietal neurons again showed the expected increased dendritic complexity and spine density. Unfortunately, our PFC results were not conclusive because we confounded the results by sex and duration of complex housing, which was only 16 days in the Comeau et al. study. Thus, it is possible that there is an initial decrease in synaptic measures over the first few days of complex housing, followed by a return to baseline levels for dendritic complexity and an increase in spine density in mPFC. There is a precedent for this type of result in studies looking at dendritic and spine changes over time after kindling of neurons in motor cortex (Teskey et al., 2001). Whatever the final story turns out to be, it is clear that the prefrontal cortex responds differently to complex housing than other neocortical regions, the hippocampus (HPC), and striatum.

One powerful sensory experience for the developing brain is tactile stimulation (e.g., Field, 2003; Guzzetta et al., 2009). Although few studies have examined the effect on prefrontal cortex, Richards et al. (2012) showed that early tactile stimulation (15 min of light stimulation with a soft brush, 3 times daily for 14 days) improves motor and cognitive functions in adulthood as well as increasing dendritic length and spine density across many forebrain structures, including in both mPFC and OFC. We are unaware of similar studies on adult rats, although Gibb et al. (2010) reported increased dendritic length in the forelimb region of the motor cortex of animals receiving tactile stimulation in adulthood. Although the prefrontal cortex was not measured, the forelimb motor cortex is adjacent to the prefrontal cortex and the study provides proof of principle that tactile stimulation alters cortical neuronal morphology in adulthood. Similarly, training rats to execute skilled forelimb movements increases spine density and dendritic length in forelimb motor cortex (e.g., Withers and Greenough, 1989; Kolb et al., 2008). But although mPFC lesions severely impair performance on the skilled reaching tasks (e.g., Whishaw et al., 1992), we are unaware of any studies reporting synaptic changes in mPFC following reach training.

## Gonadal hormones

The apparent sex differences in the effect of complex housing in the PFC suggests that there may be a fundamental difference in dendritic organization in females and males. That appears to be the case. Several studies have shown that females have shorter and less branchy dendritic arbors in mPFC than males (Kolb and Stewart, 1991; Markham and Juraska, 2002; Garrett and Wellman, 2009). Given that mPFC contains both estrogen and progesterone receptors (Pilgrim and Hutchison, 1994) it is likely that the sexual dimorphism is mediated by gonadal hormones (see review by Juraska et al., 2013). Indeed, Kolb and Stewart (1991) showed that the sex difference was absent in neonatally gonadectomized animals. In contrast to the sex difference in mPFC, in OFC the effect is reversed as females have longer and more branchy neurons than males. And, as in mPFC, this difference is abolished following neonatal gonadectomy (Kolb and Stewart, 1991).

The sexually-dimorphic effects of stress on PFC neurons suggests that there are likely to be sex differences in cognitive and/or emotional behaviors such as working memory and anxiety (see also Section Stress below). Sutcliffe et al. (2007) compared the performance of male and female rats on working and spatial memory versions of a novel object recognition task: females performed better on the first task whereas males did better on the spatial version. Similarly Johnston and File (1991) compared the anxiety-related behaviors of males and females in several paradigms (social interaction, elevated plus maze, Vogel conflict test) finding sex differences in all tests, with males generally showing higher anxiety. In fact, the authors question whether any of the tests are valid tests of anxiety in females.

## Psychoactive drugs

**Figure 2:**
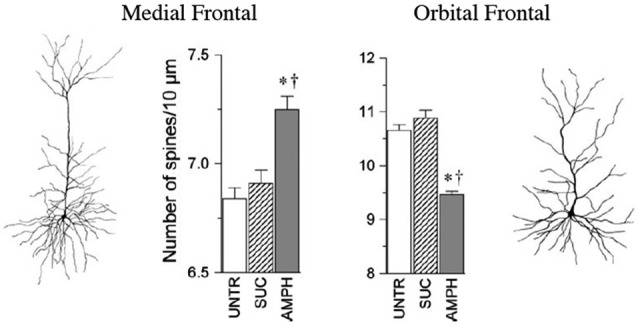
**The medial and orbital prefrontal regions show opposite chronic effects of amphetamine on spine density in adulthood**. Abbreviations: UNTR, untreated; SUC, sucrose treated; AMPH, amphetamine treated. (After Crombag et al., 2005). ^*†^ indicate a significant difference from UNTR and SUC conditions, respectively (*P* < 0.05).

Some of the largest and most robust plastics changes in prefrontal cortex are seen in the effects of repeated doses of psychoactive drugs. When animals are given repeated doses of psychomotor stimulants, there is an escalating behavioral effect (such as increasing activity to each dose) that is correlated with dendritic changes in prefrontal cortex and NAcc (e.g., Robinson and Kolb, 1999a,b, 2004). For example, when animals got repeated low doses of amphetamine given IP there was an *increase* in dendritic length and spine density in mPFC and NAcc but a corresponding *decrease* in these measures in OFC (Crombag et al., 2005; see Figure 2). Curiously, when amphetamine was injected into the ventral tegmental region, the opposite effects were observed, namely a decrease in mPFC and NAcc and an increase in OFC (Singer et al., 2009). There are now considerable data on the effects of other psychoactive drugs given in adulthood, including nicotine, morphine, phencyclidine, and THC (see Table 1).

**Table 1:** **Summary of the effects of adult exposure to psychoactive drugs on prefrontal neurons**.

	mPFC		OCF	
Drug	Dendrites	Spines	Dendrites	Spines
Amphetamine (i.v. or i.p.)	**↑**	**↑**	–	**↓**
Amphetamine in VTA	**↓**	**↓**	**↑**	**↑**
Cocaine	**↑**	**↑**	NA	–
Nicotine	**↑**	**↑**	NA	NA
Morphine	NA	**↓**	NA	**↑**
THC	**↑**	**↑**	**↓**	–
Phencyclidine	NA	**↑**	NA	NA

*Abbreviations: **↑**, increase; **↓**, decrease; –, no effect; NA, no data available*.

The key message here is that although different drugs have differing effects on prefrontal morphology, all of these drugs induce persistent changes in prefrontal neuronal structure, and in most cases the drug effects are different in mPFC vs. OFC. One caveat here is that cortical layer is important. Ferrario et al. (2005) found no effect of self-administered cocaine on layer III OFC pyramidal cells, even though layer V cells lose spines (Courley et al., 2012).

The changes in PFC neurons are rapid. Cocaine-induced increases in spine density are detectable 2 h after injection (Muñoz-Cuevas et al., 2013) and the effects of single injections of methamphetamine in adulthood or early development on neurons in mPFC can persist for months in gerbils (e.g., Dawirs et al., 1991; Blaesing et al., 2001).

The differing effects of the drugs on mPFC vs. OFC is surprising given that both regions receive similar midbrain dopaminergic projections, parallel projections from MD and amygdala, and parallel striatal connections. It appears, therefore, that different drugs reorganize these closely related prefrontal regions in very specific and very different ways. With this in mind we compared the epigenetic changes related to exposure to nicotine or amphetamine in the two prefrontal regions (Mychasiuk et al., 2013). As with the anatomical changes, the changes in gene expression in the two regions were very different. Following a two-week withdrawal period, exposure to amphetamine or nicotine was associated with a decrease in global DNA methylation in each brain region examined. Previous exposure to nicotine was associated with changes in expression of 10 genes (mPFC:5, OFC:5) whereas exposure to amphetamine was associated with changes in expression of 12 different genes (OFC:8, mPFC:4). There was no overlap in the gene expression changes in mPFC and OFC.

One important correlate of the dendritic changes in mPFC is an associated increase in Fibroblast Growth Factor-2 (FGF-2) expression (Flores et al., 1998, 2000). Although the exact role of the enhanced FGF-2 expression is uncertain, FGF-2 is proposed to reduce neuron excitability by inhibiting both voltage-gated sodium currents (Hilborn et al., 1998) and potassium currents (Cuppini et al., 2009). Thus, we might predict that the drug-associated changes in prefrontal neurons could influence the later plasticity of these neurons unless the FGF-2 levels decline (see Section Social Experience below).

Another mechanism of dendritic changes in PFC is likely related to cytoskeletal regulatory proteins. Dendritic morphology is stabilized by an underlying actin cytoskeleton. Flexibility in dendrites and spines is partly controlled by the Rho family GTPases such that an increase in Rho activation reduces dendritic growth whereas an increase promotes it (e.g., Sin et al., 2002; Sfakianos et al., 2007; Murakoshi et al., 2011). DePoy et al. (2013) showed that inhibition of Rho-kinase inhibition early in life may protect against pathological reward-seeking related to cocaine in adulthood. Differences in Rho activity may provide a mechanism for the differential effects of psychoactive drugs on the spines and dendrites of the mPFC and OFC.

The focus of most studies of drug-neuron changes in PFC have been on pyramidal neurons but it would be surprising if there were not also changes in inhibitory neurons. For example, Dawirs et al. (1997) gave gerbils a single injection of methamphetamine in adulthood and found a 20% increase in the density of GABAergic innervation in mPFC. We are unaware of Golgi-type studies of interneurons in either mPFC or OFC following exposure to psychoactive drugs but such studies would be informative given the importance of inhibition in cerebral function (e.g., Takesian and Hensch, 2013).

## Learning of neuropsychological tasks

Lesions of both the mPFC and OFC are associated with areal-specific deficits in a range of neuropsychological tasks (e.g., Kolb et al., 1974, 1994; Uylings et al., 2003; Kesner and Churchwell, 2011; Wilson et al., 2014). For example, rats with mPFC lesions are impaired at a wide range of spatial tasks, especially those requiring working memory (e.g., Kolb et al., 1983, 1994), reversal learning (e.g., de Bruin et al., 2000) or attentional shift (Birrel and Brown, 2000) whereas OFC lesions especially disrupt performance of various odor-related tasks (Otto and Eichenbaum, 1992; McDannald et al., 2014) as well associative learning (e.g., Gallagher et al., 1999; Schoenbaum et al., 2009). Given that prefrontal regions appear necessary, although likely not sufficient, to perform these tasks, it seems reasonable to expect that the acquisition of the tasks might lead to synaptic changes in one or other of the prefrontal regions. Indeed, they do.

Two studies have examined the effects learning a working memory task (delayed nonmatch-to-sample) finding changes in both mPFC and OFC (Kolb et al., 2008; Comeau et al., 2010). Both regions showed increased dendritic complexity compared to untrained yoked controls but there was an increase in spine density in mPFC and a decrease in OFC. In contrast, spatial reversal learning had a rather different effect. There was an overall decrease in mPFC and OFC neuronal branch order and length in both the trained and yoked animal groups. The same was also true for OFC spine density. Thus, it was not the training *per se* that changed the neurons but rather the mere experience of being in the maze (a Grice Box), whether the rewards were related or unrelated to behavior. Curiously, the neuronal change was a decrease in overall synapse number as reflected by reduced dendritic length and spine density.

One study has examined the effect of training in an olfactory task on functional connectivity in the OFC (Schoenbaum et al., 2000). The authors pursued the general idea that correlated activity between sets of neurons likely reflect plastic changes in the functional interactions between neurons. When rats learned an odor discrimination and subsequent reversal, there was increased correlated firing in OFC during accurate trials. It would be valuable to know if the increased correlated firing is related to changes in synaptic organization seen postmortem. It seems likely that they are but we are not aware of any direct study of this.

Taken together, the data from learning studies do not tell a simple story. It is true that tasks affected by prefrontal lesions do show changes in presumed prefrontal networks, but they are not straight forward. For example, whereas only mPFC lesions disturb working memory, OFC neurons were also changed, although in the opposite direction. Similarly, in spatial reversal learning, which is a hallmark of prefrontal injury across species (e.g., Warren, 1972) there was a reduction in synaptic space that was related not to the reversal learning but rather to the training environment. It is clear that more studies on a wider variety of behavioral tasks are required.

## Stress

Although most studies in the literature emphasize the effect of stressful experiences on the HPC, stress alters the morphology of dendritic arbor, spine, and synapse number in many brain regions, including the HPC, amygdala, and the prefrontal cortex. The stress effects are correlated with changes in cognitive function as well as emotional regulation and other self-regulatory behaviors (McEwen and Gianaros, 2011; McEwen and Morrison, 2013).

**Figure 3:**
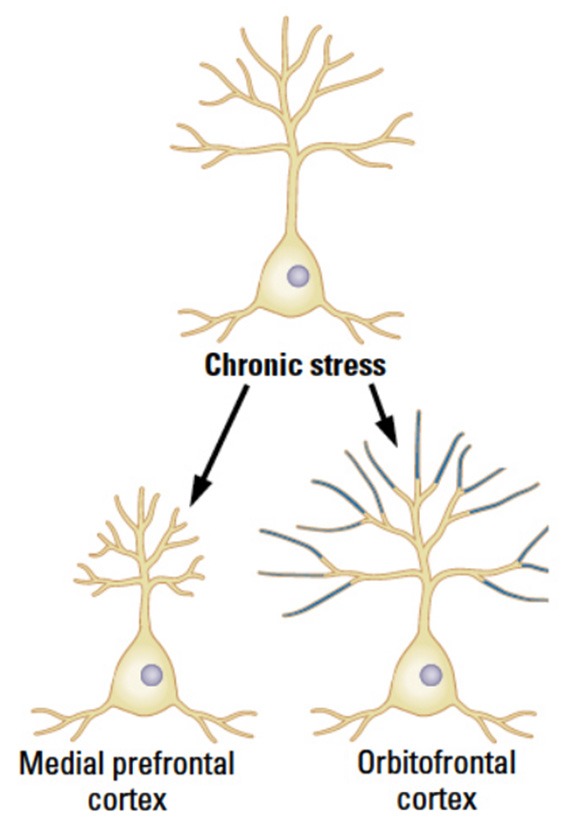
**The medial and orbital prefrontal regions show opposite chronic effects of stress in male rats in adulthood**. (After McEwen and Morrison, 2013).

Chronic stress reduces synaptic space in pyramidal neurons in layer 3 throughout the mPFC in male rats but the effects are specific to the distal portions of the apical branches with an estimated total 30% loss of axospinous synapses (Cook and Wellman, 2004; Radley et al., 2004, 2005, 2006, 2008; Bloss et al., 2010, 2011). Even though the synaptic loss is dramatic, it recovers quickly over 3 weeks in young animals, although the recovery largely occurs in the proximal rather than the distal apical regions. This recovery is not seen in older animals, however (Goldwater et al., 2009). In contrast to the changes in mPFC, the pyramidal neurons in OFC show a stress-related increase in dendritic length and spine density (Liston et al., 2006; see Figure 3). It is unknown if the OFC changes persist or shrink over time.

Although most studies of stress-related PFC changes employ chronic stress, even short-term mild stress produces dendritic retraction in mPFC (Brown et al., 2005; Izquierdo et al., 2006). The Izquierdo study compared the effects in two mPFC subregions, infralimbic (IL) and prelimbic cortex (PL) and found the mild stress effects to only be in IL although other studies have shown effects of chronic stress in PL. Further work (Lin et al., 2015) has shown that the stress effects in PL are related to the action of stress on D1 receptors. The authors make the interesting proposal that PFC D1 receptors play a central role in regulating dendritic morphology in the absence of stress.

There are sex differences in the effects of stress on mPFC pyramidal neurons. Garrett and Wellman (2009) (see review by Farrell et al., 2013) showed that whereas males show a general shortening of dendrites, females do not. The effect was estradiol dependent. Shansky et al. (2009, 2010) showed, however, that the details of the sex difference is circuit specific. They showed that in male rats mPFC neurons projecting to the basolateral amygdala (BLA) did not show the dendritic retraction whereas those projecting elsewhere did. In contrast, females showed an expansion of dendrites of the BLA-projecting neurons as long as the animals had circulating estrogen. Ovariectomized animals showed no change. mPFC neurons projecting elsewhere failed to show any dendritic changes after chronic stress whether or not females had circulating estrogen. In contrast to the effects on dendritic length, spine density was increased in both regions by stress in estrogen-intact animals (Shansky et al., 2010).

In contrast to the effects of adult stress on prefrontal neurons, Mychasiuk et al. (2011b) found that gestational stress had no chronic effect on mPFC dendritic length but decreased it in OFC in both sexes in juvenile brains. Spine density went up in both regions in both sexes. When the authors used stereological methods to estimate neuron numbers in mPFC and OFC a sex difference appeared, however. Males had significantly fewer neurons in OFC. When neuron number, dendritic length, and spine density were combined to determine a simple estimate of synapse number there was a clear sex difference related to stress. Males showed an increase in synapse number in mPFC and a decrease in OFC whereas females showed the opposite (see also Muhammad and Kolb, 2011c; Muhammad et al., 2012).

It is important to recognize that stress (and other experiences) is acting on brain regions, including prefrontal cortex, that are already sexually-dimorphic. For example, Kolb and Stewart (1991) showed that the pyramidal neurons in mPFC of males are more complex than those of females whereas the reverse pattern is true in OFC. These differences disappear in neonatally gonadectomized animals. Given that gonadal steroid receptors and actions are widespread in the brain, there are likely to be interactions between sex, stress, cognitive functions, age, and other experiences that modify prefrontal organization and function.

Finally, there is one study looking at the epigenetic effects of adult stress in mPFC, OFC, and the HPC of male and female rats (Kolb et al., 2014). There was an increase in global methylation in both sexes in both prefrontal regions but a decrease in the HPC in males but not females. When examining the RNA from the same brain regions, chronic stress exposure was associated with changes in expression of 58 genes in male rats (HPC:9, mPFC:17, OFC:32) whereas the same exposure in female rats was associated with changes in expression of 76 genes (HPC:17, mPFC:18, OFC:41). There was little overlap in gene expression by sex or region. These epigenetic data are consistent with the morphological data in showing both regional and sex differences in the effect of stress on the prefrontal cortex, and, in addition, demonstrate that the effects are different than those seen in the HPC. Similar results are found for epigenetic changes following gestational stress as well (Mychasiuk et al., 2011a,b).

## Social experience

The prefrontal cortex of a wide range of mammals has been shown to play a role in the control of social behavior. Thus, lesions to either the mPFC or OFC in rats lead to changes in both adult social and juvenile play behavior (e.g., Kolb, 1974; de Bruin, 1991; Pellis et al., 2006). Manipulations of these behaviors modify prefrontal pyramidal cell morphology.

In one set of studies (Hamilton et al., 2004; Silasi et al., 2008), rats were given a changing social milieu by a schedule of rotating cage partners. Rats in the changing social group received a new cage and bedding as well as a new cage partner every 48 h. Animals in the no-treatment groups only received a new cage and bedding. Although social animals received a different cage partner every 48 h, the animals were generally familiar with one another as they had been housed together for 18 days prior to switching, and rats are known to be capable of maintaining social memories for many weeks (Galef and Whiskin, 2003). Every fifth switch resulted in the original cage partners being matched, from which point the original switching cycle was repeated for two cycles. The social experience increased dendritic length in the OFC but had little effect in the mPFC.

One important type of social experience in all mammals is play. Bell et al. (2010) manipulated the amount of play behavior that rats could experience by housing them with either 1 or 3 siblings or 1 or 3 adults. Adult rats do not play with juveniles or adolescents so although there would be social interaction if youngsters were housed with adults, there is virtually no play. Furthermore, the more siblings living together, the more play there is. Bell et al found that the amount of play was negatively correlated with neuronal complexity: the more play there was, the fewer spines in both mPFC and OFC, the difference being in the range of 10–20%. This drop in spine density is surprising given that Helmeke et al. (2009) showed that paternal deprivation during infancy in degus produced a dramatic drop in spine density in OFC, the effect being about 50%. The effects in the Helmeke study were taken as negative whereas the effects in the Bell study were presumed to be positive and related to enhanced subsequent plasticity (see Section Social Experience below). The difference could be related either to the large difference in magnitude of spine loss or the developmental age of the experience.

## Metaplasticity

To this point we have focused on how singular changes induce plastic changes in PFC. But life is more complex than single experiences—we have one experience after another. Abraham and Bear (1996) coined the term “metaplasticity” to refer to the idea that plastic changes in the brain will modulate how the brain changes in response to subsequent experiences. There have not been extensive studies on metaplastic changes in PFC but several studies suggest this to be a fruitful route of study. There are two general types of study: serial experiences in adulthood vs. early-life experiences followed by other experiences in adulthood. We consider each separately.

*Serial experiences in adults*. In two separate studies rats were sensitized to either amphetamine, cocaine, or nicotine for 20 days (amphetamine), 28 days (cocaine), or 14 days (nicotine) (Kolb et al., 2003a; Hamilton and Kolb, 2005). The animals then were placed in complex environments (or group-housed in lab cages) for 3 mo. The morphological effects of the drugs were still evident after the 3 mo of complex housing, but surprisingly, the prior exposure to the drugs blocked the effects of complex housing in both NAcc and parietal cortex (Kolb et al., 2003a; Hamilton and Kolb, 2005). Given the robustness of complex-housing effects, this is a surprising result. Indeed, given that the parietal cortex does not show a synaptic change in response to the drugs, the failure of the housing to alter the parietal neurons is unexpected. Although the authors did not report data from PFC in these studies, we would anticipate that the drugs would also block the changes related to complex housing there as well.

A reasonable question arising from the drug/housing studies is whether complex housing would alter the effect of drugs given *after* the housing experience. Hamilton and Kolb (2005) found no effect of the housing experience on later sensitizing doses of nicotine. This result may not mean that the experiences did not interact in affecting cortical plasticity but at least in the measures used in the study there was no effect.

The mechanism(s) underlying the reduced plasticity following drug exposure could be related to either the epigenetic changes or the FGF-2 related changes in neuron excitability (see References above). Both complex housing and psychomotor stimulants (Kolb et al., 2014) induce epigenetic changes so it is possible that these changes interact to make the PFC neurons less plastic. Similarly, the increased FGF-2 expression associated with psychomotor stimulants may decrease neuron excitability and interfere with the experience-dependent changes. Another factor here is that complex housing also increases FGF-2 (Kolb et al., 1997), which might predict a reverse effect of complex housing on the drug-induced neuronal changes. Given that we do not know if nicotine also increases FGF-2 the negative finding of the Hamilton and Kolb (2005) study could be because nicotine does not increase FGF-2.

*Early experiences followed by adult experiences*. Following from the Hamilton and Kolb study showing no effect of housing on later effects of nicotine, Li et al. (2010) showed that being raised in a complex environment from conception to adulthood significantly attenuated, but did not eliminate, the effect of amphetamine both on behavioral sensitization and the drug-induced effects in both mPFC and OFC. It is not clear if the difference is in the drug (nicotine vs. amphetamine) or the age at which the complex experience began.

In a series of studies Muhammad and Kolb (2011a,b,c) and Muhammad et al. (2011) examined the effects of perinatal experiences on the adult response to amphetamine. Both prenatal (gestational) and infant (first two weeks of life) tactile stimulation significantly reduced the amphetamine-induced behavioral sensitization as well as the drug-induced structural changes in PFC. In contrast, prenatal (gestational) stress or maternal separation in infancy had no effect on later amphetamine-induced behavioral sensitization. Gestational stress also failed to influence the drug-induced changes in PFC neurons. To our surprise, however, whereas maternal separation alone increased spine density in both PFC regions, maternal separation also blocked the expected drug-induced changes in spine density in both regions. It is not known why the two types of stressors had such different effects but a later study directly comparing the effects of the two stressors showed that although both experiences altered dendritic morphology, they did so in different ways (Muhammad et al., 2012). The different developmental stressors are affecting the brain at different stages of development, which evidently makes a large difference to PFC organization. At any rate, the key point of this group of studies is that early experiences not only alter PFC development (for a review, see Kolb et al., 2012) but also interact with later behavioral and anatomical effects of psychomotor stimulants.

Finally, there are studies examining the effect of the amount of play behavior in the juvenile period on both PFC regions and how this might interact with later exposure to nicotine. As noted above, Bell et al. (2010) first showed that juvenile play experience altered the pruning of PFC neurons: mPFC and OFC pyramidal cells had a reduced spine density in rats with extensive play behavior. In a follow-up study Himmler et al. (2013) demonstrated that later adult exposure to nicotine had a significantly increased effect on neurons in both mPFC and NAcc suggesting that one effect of play is to make these regions more plastic to later experiences. One provocative implication of this finding is that experiences that reduce play behavior, such as autism or early drug exposure, might be expected render the PFC less plastic in adulthood.

One mechanism for this reduced plasticity could be related to the projections of the dopaminergic neurons to the prefrontal cortex. The dopaminergic innervation begins in development and is ongoing until adulthood. Although the projections are relatively sparse, prefrontal neurons are highly responsive to the modulatory effects of dopamine in adulthood. Conditions such as schizophrenia, depression, and drug abuse, which usually begin to appear in adolescence, are associated with subtle alterations in mPFC circuitry and dopaminergic activity. DCC, the receptor for the guidance cue netrin-1, organizes mPFC wiring during adolescence (Manitt et al., 2011) and it is hypothesized that variations in *dcc* may influence predisposition of mPFC to behavioral disorders in adulthood (Manitt et al., 2013). Although still speculative, we suggest that variations in DCC may be related to early experiences, which alter the prefrontal response to a range of experiences in adulthood.

## Lesion-induced changes

Although there is an extensive literature on the effects of PFC lesions both in development and adulthood on synaptic organization of the intact cortex (e.g., Kolb and Gibb, 1990, 1991), much less is know about the effects of lesions of other regions on the PFC. Perhaps the first such evidence came from the Lipska and Weinberger’s extensive studies showing that that neonatal hippocampal injury produces behaviors in adulthood that are reminiscent of behaviors seen in animals sensitized to psychomotor stimulants (for a review, see Lipska and Weinberger, 2000). Later studies revealed that there was a reduction in spine density in mPFC and an increase in spine density in the OFC (Gorny et al., 2001; Flores et al., 2005).

We are aware of only study looking at the effects of adulthood brain injury on prefrontal neurons. Gonzalez and Kolb (2003) made unilateral lesions of the motor cortex using different etiologies (suction, devascularization, vascular occlusion) to examine motor behaviors and synaptic changes in the motor cortex in the intact hemisphere and in mPFC in both the intact and injured hemisphere. Although the results varied somewhat with etiology, the general finding was that whereas mPFC neurons showed reduced dendritic complexity and spine density in the damaged hemisphere, there was an increase in the intact hemisphere. The changes in mPFC were presumed to be related to the direct cortico-spinal connections from mPFC but this idea was not tested directly.

In view of the extensive connections of virtually all cortical regions to the prefrontal cortex (see review by Kolb and Whishaw, 2015), it is surprising that there are not more studies looking at how extraprefrontal lesions alter prefrontal architecture and function. This would seem to be a potentially rich research pasture.

## Conclusions

Perhaps the most general conclusion we can reach is that the prefrontal cortex is extremely plastic and that the mPFC and OFC regions frequently respond very differently to the same experience in the same brain. Although this review has focused on synaptic changes (dendritic morphology and spine density) in pyramidal neurons, the literature on plastic changes in other cortical regions has shown that nearly every component of the nervous system exhibits robust, reproducible responses to experience (Markham and Greenough, 2004). Thus, not only are there changes in synaptogenesis and dendritic organization, non-neuronal components such as increased myelination, angiogenesis, astrocytic hypertrophy and astrocytegenesis also change. Different experiences drive region-specific changes and influence the stability of the changes. As a general rule of thumb, changes in synapse number and structure as well as myelination may be permanent and are learning-driven whereas changes in cerebrovasculature and astrocytes are more transient and driven by neuron activity (Markham and Greenough, 2004). There is a vast area of ignorance regarding the nature such changes in PFC, however.

We must emphasize that changes in synaptic organization (or non-neuronal components) is not done in isolation. These changes are related to behavioral changes and these changes are not unidirectional. Thus, as behavior changes, such as when animals learn new tasks, there are associated neuronal changes that may result from the behavioral changes, rather than causing them. This complicates the study of prefrontal plasticity considerably. For example, we have seen that psychoactive drugs alter PFC neurons but the drugs also change behavior so what is actually causing the neuronal changes—the drugs or the behavior? We have seen too that there are associated changes in neurotrophic factors such as FGF-2, epigenetic changes, changes in neuron excitability, D1 receptors, and so on. What role do these factors play in driving the behavioral and neuronal changes? We have also seen that experiences can interact to produce unexpected plastic changes.

Although we have not attempted to review the effects of experience on non prefrontal cortical regions, it is clear that the factors changing the prefrontal cortex often have little effect on other cortical regions, perhaps the best examples being psychoactive drugs and stress. In addition, when experiences do affect other cortical regions, the effects are often different, a prime example being complex housing. One exception is tactile stimulation, which appears to have a general effect across the cortical mantle.

Our emphasis here has been on the effects of adult experiences on PFC and behavior but there is another story to be told regarding changes related to developmental experiences, including preconceptual (Mychasiuk et al., 2013), as well as gestational, infant, and juvenile experiences (Kolb et al., 2012, 2013). Clearly, we are just beginning to understand plasticity and the prefrontal cortex.

## Conflict of interest statement

The authors declare that the research was conducted in the absence of any commercial or financial relationships that could be construed as a potential conflict of interest.
